# Increased risk of osteoporosis in patients with primary biliary cirrhosis

**DOI:** 10.1371/journal.pone.0194418

**Published:** 2018-03-15

**Authors:** Chen-Yi Liao, Chi-Hsiang Chung, Pauling Chu, Kuang-yu Wei, Tseng-Min Feng, Fu-Huang Lin, Chang-Huei Tsao, Chia-Chao Wu, Wu-Chien Chien

**Affiliations:** 1 Division of Nephrology, Department of Internal Medicine, Kaohsiung Armed Forces General Hospital, Kaohsiung, Taiwan; 2 Division of Nephrology, Department of Internal Medicine, Tri-Service General Hospital, National Defense Medical Center, Taipei, Taiwan; 3 School of Public Health, National Defense Medical Center, Taipei, Taiwan; 4 Taiwanese Injury Prevention and Safety Promotion Association, Taipei, Taiwan; 5 Department of Medical Research, Tri-Service General Hospital, National Defense Medical Center, Taipei, Taiwan; 6 Department of Microbiology & Immunology, National Defense Medical Center, Taipei, Taiwan; 7 Graduate Institute of Life Sciences, National Defense Medical Center, Taipei, Taiwan; Augusta University, UNITED STATES

## Abstract

**Background:**

We evaluated the risk of osteoporosis in patients with primary biliary cirrhosis (PBC) using a nationwide population-based dataset.

**Methods:**

In a cohort study of 986,713 individuals, we selected 2,493 PBC patients who were aged 18 years or older and had been diagnosed with PBC, based on the International Classification of Disease (ICD-9-CM) codes 571.6, during 20002010. The control cohort comprised 9,972 randomly selected, propensity matched patients (by age, gender, and index date), without PBC. Using this adjusted data, a possible association between PBC and the risk of developing osteoporosis was estimated using a Cox proportional hazard regression model.

**Results:**

During the follow-up period, osteoporosis was diagnosed in 150 (6.02%) patients in the PBC cohort and in 539 (5.41%) patients in the non-PBC cohort. After adjusting for covariates, osteoporosis risk was found to be 3.333 times greater in the PBC cohort than in the non-PBC cohort when measured over 6 years after PBC diagnosis. Stratification revealed that the use of ursodeoxycholic acid (UDCA) had no significance in decreasing the risk of osteoporosis when comparing the PBC cohorts with the non-PBC cohorts (P = 0.124). Additionally, osteoporosis risk was significantly higher in PBC patients with steroid use (aHR: 6.899 vs 3.333). Moreover, when comparing the PBC cohorts to the non-PBC cohorts, the non-cirrhotic patients were prone to osteoporosis at a younger age compared to those in the cirrhotic cohorts. We also found that the associated risk of fractures is only prominent for vertebral and wrist fractures in the PBC cohort compared to that in the non-PBC cohort.

**Conclusion:**

A significant association exists between PBC and subsequent risk for osteoporosis. Therefore, PBC patients, particularly those treated with steroids, should be evaluated for subsequent risk of osteoporosis.

## Introduction

Primary biliary cirrohsis (PBC) is a chronic and progressive cholestatic liver disease of presumed autoimmune pathogenesis that usually affects middle-aged women, which eventually leads to liver failure and the need for liver transplantation. Typically, PBC is characterized by non-extrahepatic biliary obstruction, increased alkaline phosphatase levels, the presence of increased anti-mitochondrial antibodies and histological features of non-suppurative destructive cholangitis along with the destruction of interlobular bile ducts.

Osteoporosis is characterized by loss of bone strength caused by dramatically attenuated bone mineral density (BMD), reduced by at least 2.5 standard deviations from the peak bone density of healthy young subjects, and by compromised bone quality, which results in high susceptibility to fragility fractures. The complications associated with osteoporosis can substantially burden affected individuals, their families, and the health care system. Therefore, the relevant risk factors should be identified to reduce the burden.

Disorders of the liver and the gastrointestinal tract, particularly chronic inflammatory process such as PBC, are commonly associated with osteopenia and osteoporosis.

However, controversy exists as to whether people with primary biliary cirrhosis (PBC) have an increased risk of developing osteoporosis fractures.[1]The reported prevalence of osteoporosis among patients who had PBC varies remarkably from 20% to 90%.[2,3]Some researchers have found that PBC is associated with an increased risk of osteoporosis,[4,5]but others have not.[6,7]Nonetheless, it is clear that the prevalence increases with disease progression, and up to 80% of patients with cirrhosis indeed have osteoporosis.[8]Remarkably, only a few studies have discussed the relationship between osteoporosis and cirrhotic as well as non-cirrhotic PBC patients,[9]and only 2 have evaluated the overall fracture risk in people with PBC compared with the general population.[10,11]

The possible association between the use of steroids and PBC has not been fully resolved. Therefore, we performed a population cohort study using the NHIRD (National Health Insurance Research Database) to quantify the excess fracture risk and evaluate the risk of osteoporosis with and without steroid use in people with PBC. We additionally sought to clarify the risk of osteoporosis in PBC patients with and without liver cirrhosis.

## Material and methods

### Data source

The National Health Insurance (NHI) program in Taiwan is a compulsory single-payer program initiated in March 1995. Currently, there are 23.75 million enrollees, representing virtually the entire (99.9%) population of the country. The National Health Research Institutes maintain the NHIRD, containing all claims data. Herein, we used the NHIRD inpatient and outpatient datasets and the Registry of Beneficiaries. Diagnoses in the database were based on the International Classification of Disease, Ninth Revision, Clinical Modification (ICD-9-CM) codes. Patient confidentiality was ensured by double-encrypted identifiers in the NHIRD.

### Study participants

**Fig 1:**
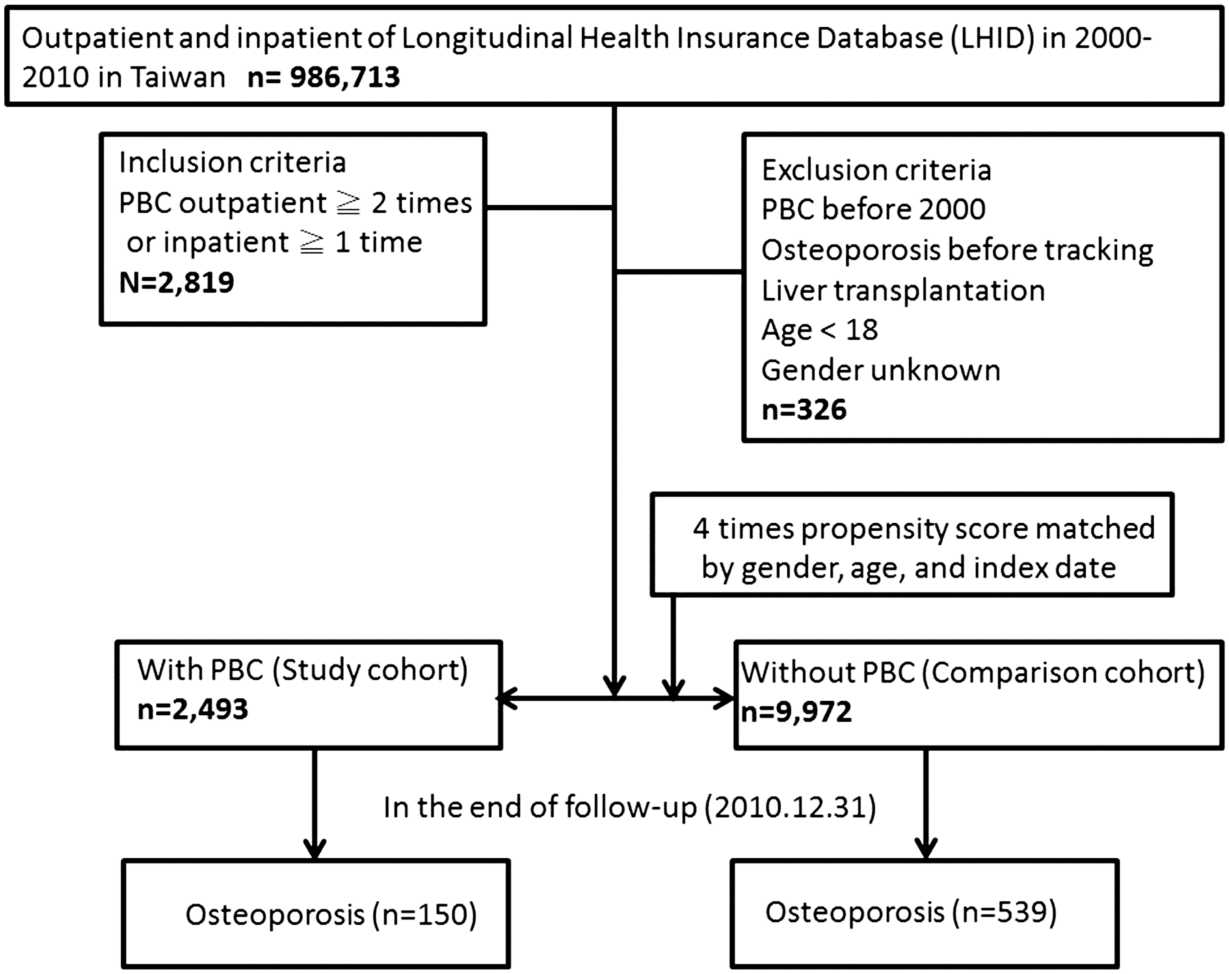
The flowchart of study sample selection from National Health Insurance Research Database in Taiwan.

The study design and specific patient characteristics, with details of inclusion and exclusion criteria, are shown in Fig 1. The control cohort (non-PBC patients) was randomly matched with PBC patients according to age, sex, and index date (four controls for each PBC patient) using the same exclusion criteria. The study cohort included 986,713 patients aged 18 years and older who had been diagnosed with PBC (ICD-9-CM codes 571.6) during 2000–2010. The index date was designated as the first clinical visit for PBC. The exclusion criteria were as follows: diagnosis with PBC before 2000, osteoporosis (ICD-9-CM code 733) before the index date, incomplete data, status of post liver transplantation, aged below 18 years, and unknown gender. The ratio of PBC patients to non-PBC patients was maintained at 1:4 to enhance the power of the statistical tests employed, particularly regarding stratification analysis. Using these criteria, 9,972 non-PBC patients were identified.

### Outcome

Patients in both the PBC and non-PBC cohort*s* were followed up until the end of 2010 or until one of the following events occurred: diagnosis with osteoporosis, elimination because of lack of follow-up, withdrawal from insurance, or death. Herein, we examined the impact of numerous baseline comorbidities and baseline sociodemographic characteristics including age, sex, and urbanization level, as well as the Charlson comorbidity index (CCI) score of the PBC cohort, and we adjusted the environmental factors according to urbanization level. The analysis also included the use of corticosteroids.

### Statistical analysis

Baseline distributions of demographic characteristics and comorbidities were compared between PBC and non-PBC patients using the χ2 test for categorical variables and the *t*-test for continuous variables. The incidence density of osteoporosis (per 10^5^ person-years) was calculated in both cohorts. Additionally, we calculated the incidence rate ratio (IRR) of osteoporosis for each variable. Univariate and multivariate Cox proportional hazards regression models were used to examine the influence of PBC on the risk of osteoporosis, which was expressed as a hazard ratio (HR) with a 95% confidence interval (CI), using non-PBC patients as the reference. Multivariate models were controlled for a considerable range of independent variables: age, sex, hypertension, diabetes mellitus, CHF, stroke, CAD, hyperlipidemia, dementia, steroid use, long-term bedridden, alcohol attributed disease, tobacco use disorder, obesity, postmenopausal, CKD, hyperparathyroidism, COPD, RA, hyperthyroidism, celiac disease, IBD, Sjogren’s syndrome, renal tubular acidosis, liver cirrhosis, CCI score, season, city location, urbanization, and level of care. After stratifying by age, sex, comorbidities, and follow-up time, the relative risk for osteoporosis in the PBC cohort was compared with that for non-PBC cohorts using the Cox model. Cumulative incidence curves of osteoporosis for the two cohorts were assessed using the Kaplan–Meier analysis; differences between cohorts were evaluated using the log-rank test. All data were analyzed using SAS statistical software (Version 9.3 for Windows). A two-tailed P < 0.05 was considered significant.

### Ethics statement

We employed the NHIRD encrypted patient personal information system to protect patient privacy; therefore, patient consent was not required for us to access the NHIRD. This study was approved by the Institutional Review Board of the Tri-Service General Hospital. (TSGHIRB No. 2-105-05-082).

## Results

During 2000–2010, a total of 2,819 PBC patients were enrolled in this study in accordance with our inclusion criteria; osteoporosis was observed in 150 of 2,493 PBC patients and in 539 of 9,972 non-PBC patients.

Table 1 lists demographic characteristics and comorbidities of the PBC (2,493) and non-PBC cohorts (9,972) during 2000–2010. In both cohorts approximately 49% were more than 60 years of age, 56% were women, and the proportion by age and sex were similar. The PBC cohorts had a higher proportion of individuals living in urban areas (38.83% vs 33.71%; P < 0.001). The following comorbidities were significantly more likely in the PBC than in the baseline non-PBC cohort: LC (11.39% vs 2.68% P < 0.001), hyperthyroidism (0.68% vs 0.30%; P = 0.007), Sjogren’s syndrome (2.13% vs 0.04%; P < 0.001), and renal tubular acidosis (2.25% vs 0.95%; P < 0.001). The PBC cohort had a higher distribution in the summer (24.83% vs 23.87%) and autumn (24.47% vs 21.89%; P = 0.008) and among people living in northern Taiwan. (49.78% vs 39.09%; P < 0.001).

**Table 1:** Demographic characteristics and comorbidities in PBC and non-PBC cohorts.

	Total	PBC		
Variables		Yes	No	P -value
Osteoporosis patients, n (%)	12,465	2,493 (20.00)	9,972 (80.00)	
Age, year				0.999
18–29	360 (2.89)	72 (2.89)	288 (2.89)	0.999
30–39	790 (6.34)	158 (6.34)	632 (6.34)	
40–49	2,205 (17.69)	441 (17.69)	1,764 (17.69)	
50–59	3,005 (24.11)	601 (24.11)	2,404 (24.11)	
≧60	6,105 (48.98)	1,221 (48.98)	4,884 (48.98)	
Sex				0.999
Female	7,100 (56.96)	1,420 (56.96)	5,680 (59.96)	
Male	5,365 (43.04)	1,073 (43.04)	4,292 (43.04)	
Comorbidity				
LC	551 (4.42)	284 (11.39)	267 (2.68)	<0.001
Hypertension	2,056 (16.49)	266 (10.67)	1,790 (17.95)	<0.001*
DM	1,942 (15.58)	398 (15.96)	1,544 (15.48)	0.287
CHF	331 (2.66)	45 (1.81)	286 (2.87)	0.002*
Stroke	802 (6.43)	51 (2.05)	751 (7.53)	<0.001*
CAD	1,009 (8.09)	88 (3.53)	921(9.24)	<0.001*
Hyperlipidemia	361 (2.90)	70 (2.81)	291 (2.92)	0.410
CKD	602 (4.83)	90 (3.61)	512 (5.13)	0.001*
Obesity	2 (0.002)	0 (0.00)	2 (0.02)	0.640
COPD	786 (6.31)	76 (3.05)	710(7.12)	<0.001*
Dementia	134 (1.08)	23 (0.92)	111(1.11)	0.237
Postmenopausal	23 (0.18)	0 (0.00)	23 (0.23)	0.006*
Hyperthyroidism	47 (0.38)	17 (0.68)	30 (0.30)	0.007*
RA	47 (0.38)	10 (0.40)	37 (0.37)	0.471
Sjögren’s syndrome	57 (0.46)	53 (2.13)	4 (0.04)	<0.001*
Renal tubular acidosis	151 (1.21)	56 (2.25)	95 (0.95)	<0.001*
Vit D deficiency	0 (0.00)	0 (0.00)	0 (0.00)	0
Long-term bedridden	0 (0.00)	0 (0.00)	0 (0.00)	0
Hyperparathyroidism	1 (0.001)	0 (0.00)	1 (0.001)	0.800
Alcohol attributed disease	50 (0.40)	7 (0.28)	43 (0.43)	0.190
Tabacco attribuated disease	4 (0.03)	7 (0.28)	43 (0.43)	0.190
Celiac disease	0 (0.00)	0 (0.00)	0 (0.00)	0
IBD	11(0.09)	3 (0.12)	8 (0.08)	0.383
Medication, n (%)				
Steroid use	82 (0.66)	19 (0.76)	63 (0.63)	0.280
UDCA use	1,037 (8.32)	486 (19.49)	551 (5.53)	<0.001*
Denosumab use	1,413 (11.34)	298 (11.95)	1,115 (11.18)	0.274
SERMs	1,124 (9.02)	213 (8.54)	911 (9.14)	0.369
Bisphosphonate use	485 (3.89)	85 (3.41)	400 (4.01)	0.183
Relevant fracture, n (%)				
Hip fracture	133 (1.07)	6 (0.24)	127 (1.27)	<0.001*
Wrist fracture	126 (1.01)	1 (0.04)	125 (1.25)	<0.001*
Vertebral fracture	84 (0.67)	1 (0.04)	83 (0.83)	<0.001*
Rib fracture	59 0.47)	1 (0.04)	58 (0.58)	<0.001*
CCI_R	0.45±1.82	0.51±1.84	0.44±1.81	0.098
Season				0.008*
Spring (March-May)	3,377 (27.09)	652 (26.15)	2,725 (27.33)	
Summer (June-August)	2,999 (24.06)	619 (24.83)	2,380 (23.87)	
Autumn (September-November)	2,793 (22.41)	610 (24.47)	2,183 (21.89)	
Winter (December-February)	3,296 (24.44)	612 (24.55)	2,684 (26.92)	
Location				<0.001*
Northern Taiwan	5,139 (41.23)	1,241 (49.78)	3,898 (39.09)	
Middle Taiwan	3,420 (27.44)	559 (22.42)	2,861 (28.69)	
Southern Taiwan	3,139 (25.18)	559 (22.42)	2,580 (25.87)	
Eastern Taiwan	719 (5.77)	127 (5.09)	592 (5.94)	
Outlets Islands	48 (0.39)	7 (0.28)	41 (0.41)	
Urbanization level				<0.001*
1 (The highest)	4,330 (34.74)	968 (38.83)	3,362 (33.71)	
2	5,344 (42.87)	1,077 (43.20)	4,267 (42.79)	
3	871 (6.99)	102 (4.09)	769 (7.71)	
4 (The lowest)	1,920 (15.40)	346 (13.88)	1,574 (15.78)	
Level of care				<0.001*
Medical center	4,595 (36.86)	1,178 (47.25)	3,417 (34.27)	
Region hospital	4,196 (33.66)	833 (33.41)	3,363 (33.72)	
Local hospital	3,674 (29.47)	482 (19.33)	3,192 (32.01)	

Chi-square/Fisher exact test; continue variable: t-test.

*P-value <0.05.

LC denotes liver cirrhosis. DM denotes diabetes mellitus. CHF denotes congestive heart failure. CAD denotes coronary artery disease. CKD denotes chronic kidney disease. COPD denotes chronic obstructive pulmonary disease. RA denotes rheumatoid arthritis. IBD denotes inflammatory bowel disease. UDCA denotes ursodeoxycholic acid. SERMs denotes selective estrogen receptor modulators. CCI_R denotes Charlson comorbidity index removed DM, CHF, stroke, COPD, and liver diseases.

The following comorbidities were significantly more likely in the non-PBC than in the PBC cohort: hypertension (17.95% vs 10.67%; P < 0.001), congestive heart failure (2.87% vs 1.81%; P = 0.002), stroke (7.53% vs 2.05%; P < 0.001), CAD (9.24% vs 3.53%; P < 0.001), CKD (5.13% vs 3.61%; P = 0.001), COPD (7.12% vs 3.05%; P < 0.001), postmenopausal (0.23% vs 0.00%; P = 0.006), hip fracture (1.27% vs 0.24%; P < 0.001), wrist fracture (1.25% vs 0.04%; P < 0.001), vertebral fracture (0.83% vs 0.04%; P < 0.001), rib fracture(0.58% vs 0.04%; P < 0.001). Notably, anti-resorptive agents including denosumab, selective estrogen receptor modulators (SERMs) and bisphosphonate) and steroid use in both cohorts showed no significant difference (0.76% vs 0.63%; P = 0.28).

### Osteoporosis incidence and risk

Table 2 presents the demographic data of the PBC and non-PBC cohorts after 10 years of follow-up. During this period osteoporosis was significantly higher in the PBC cohorts than in the non-PBC cohorts (6.02% vs 5.41%; P = 0.024). The PBC cohort was significantly younger than the non-PBC cohort (mean age: 61.12 ± 14.98 vs 63.41 ± 15.50; P = 0.001). Additionally, the PBC cohort was significantly higher in patients with Sjogren’s syndrome than the non-PBC cohort (1.89% vs 0.10%; P < 0.001). Similarly, ursodeoxycholic acid (UDCA) use was significantly higher in the PBC cohort (19.49% vs 5.53%; P < 0.001). Perhaps, UDCA could be used in the non-PBC cohort owing to its role in dissolution of small- to medium-sized radiolucent, cholesterol-rich gallstones in patients with a functioning gallbladder. Conversely, the PBC cohort had significantly fewer patients with hypertension; DM; CHF; stroke; CAD; hyperlipidemia; COPD; dementia; and hip, wrist, vertebrae, and rib fractures, than the non-PBC cohort. However, the PBC cohort was significantly more represented in autumn (31.61% vs 25.52%), living in northern Taiwan (47.93% vs 38.93%), living in the highest urbanization place (38.07% vs 32.51%), and having been diagnosed in a medical center (46.77% vs 35.20%), than the non-PBC cohort.

**Table 2:** Demographic characteristics and comorbidities in PBC and non-PBC cohorts after 10 years follow up.

	Total	PBC		
Variables		Yes	No	P -value
Osteoporosis patients, n (%)	689	150 (6.02)	539 (5.41)	0.024*
Age, year				0.001*
18–29	257 (2.06)	58 (2.33)	199 (2.00)	0.001*
30–39	681 (5.46)	5133(5.33)	548 (5.50)	
40–49	1,680 (13.48)	406 (16.29)	1,274 (12.78)	
50–59	2,634 (21.13)	604 (24.23)	2,030 (20.36)	
≧60	7,216 (57.87)	1,292 (51.83)	5,921 (59.38)	
Sex				0.999
Female	7,100 (56.96)	1,420 (56.96)	5,680 (59.96)	
Male	5,365 (43.04)	1,073 (43.04)	4,292 (43.04)	
Comorbidity				
LC	1,182(9.48)	738 (29.60)	444 (4.45)	<0.001*
Hypertension	2,267 (18.19)	288 (11.55)	1,979 (19.85)	<0.001*
DM	2,222 (17.83)	406 (16.29)	1,816(18.21)	0.013*
CHF	576 (4.62)	59 (2.37)	517 (5.18)	<0.001*
Stroke	936 (7.51)	584 (3.37)	852 (8.54)	<0.001*
CAD	1,022 (8.20)	105 (4.21)	917 (9.20)	<0.001*
Hyperlipidemia	327 (2.62)	51 (2.05)	276 (2.77)	0.026*
CKD	996 (7.99)	196 (7.86)	800 (8.02)	0.412
COPD	884 (7.09)	88(3.53)	796 (7.98)	<0.001*
Dementia	186 (1.49)	23 (0.92)	163(1.63)	<0.001*
Sjögren’s syndrome	57 (0.46)	47 (1.89)	10 (0.10)	<0.001*
Medication, n (%)				
Steroid use	117 (0.94)	24 (0.96)	93 (0.93)	0.491
UDCA use	1,037(8.32)	486(19.49)	551(5.53)	<0.001*
Denosumab use	1,567 (12.57)	333 (13.36)	1,234 (12.37)	0.188
SERMs	1,272(10.20)	259 (10.39)	1,013 (10.16)	0.739
Bisphosphonate use	613 (4.92)	117 (4.69)	496 (4.97)	0.605
Relevant fracture, n (%)				
Hip fracture	141 (1.13)	18 (0.72)	123 (1.23)	0.020*
Wrist fracture	102(0.82)	7 (0.28)	95 (0.95)	0.001*
Vertebral fracture	117 (0.94)	7 (0.28)	110 (1.10)	<0.001*
Rib fracture	62 0.50)	3 (0.12)	59 (0.59)	0.001*
CCI_R	0.86±2.77	0.81±2.50	0.87±2.84	0.350
Season				<0.001*
Spring (March-May)	2,966 (23.79)	500 (20.06)	2,466 (24.73)	
Summer (June-August)	3,073 (24.65)	605 (24.27)	2,468 (24.75)	
Autumn (September-November)	3,333 (26.74)	788 (31.61)	2,545 (25.52)	
Winter (December-February)	3,093 (24.81)	600 (24.07)	2,493 (25.00)	
Location				<0.001*
Northern Taiwan	5,077 (40.73)	1,195 (47.93)	3,882 (38.93)	
Middle Taiwan	3,445 (27.64)	578 (23.18)	2,867 (28.75)	
Southern Taiwan	3,150 (25.27)	5573(22.98)	2,577 (25.84)	
Eastern Taiwan	736 (5.90)	137 (5.50)	599 (6.01)	
Outlets Islands	57 (0.46)	10 (0.40)	47 (0.47)	
Urbanization level				<0.001
1 (The highest)	4,191 (33.62)	949(38.07)	3,242 (32.51)	
2	5,456(43.77)	1,076 (43.16)	4,380 (43.92)	
3	851 (6.83)	103 (4.13)	748 (7.50)	
4 (The lowest)	1,967 (15.78)	365 (14.64)	1,602 (16.06)	
Level of care				<0.001
Medical center	4,676 (37.51)	1,166 (46.77)	3,510 (35.20)	
Region hospital	4,849 (38.90)	946 (37.95)	3,903 (39.14)	
Local hospital	2,940 (23.59)	381 (15.28)	2,559 (25.66)	

Chi-square/Fisher exact test; continue variable: t-test.

*P-value <0.05

LC denotes liver cirrhosis. DM denotes diabetes mellitus. CHF denotes congestive heart failure. CAD denotes coronary artery disease. CKD denotes chronic kidney disease. COPD denotes chronic obstructive pulmonary disease. UDCA denotes ursodeoxycholic acid. SERMs denotes selective estrogen receptor modulators. CCI_R denotes Charlson comorbidity index removed DM, CHF, stroke, COPD, and liver diseases.

Several multivariate analyses with adjustments for age, sex, and comorbidities reported in Table 3 revealed that osteoporosis risk was a remarkable 3.333 times greater in the PBC cohort than in the non-PBC cohort (aHR: = 3.333, 95% CI = 2.712–4.098, P < 0.001) after adjusting for age, CCI, related comorbidities (hypertension, diabetes mellitus, CAD, hyperlipidemia, chronic kidney disease, COPD, hyperthyroidism, hyperparathyroidism, RA, celiac disease, IBD, stroke, Sjogren’s syndrome, and RTA), the use of medication corticosteroids, UDCA, anti-resorptive agents including denosumab, SERMs and bisphosphonate), obesity, postmenopausal, long-term bedridden, tobacco use disorder, and alcohol attributed disease. Additionally, we observed that osteoporosis risk was higher in RA (aHR = 2.111, 95% CI = 1.301–3.989, P < 0.001), hip fracture (aHR = 2.267, 95% CI = 1.542–3.796, P < 0.001), and vertebral fracture (aHR = 6.904, 95% CI = 5.101–8.999, P < 0.001). Conversely, the osteoporosis risk was lower in males (aHR = 0.567, 95% CI = 0.419–0.678, P < 0.001) and hypertension (aHR = 0.611, 95% CI = 0.436–0.786, P<0.001), CHF (aHR = 0.499, 95% CI = 0.312–0.795, P = 0.001), CKD (aHR = 0.459, 95% CI = 0.317–0.754, P < 0.001), and RTA (aHR = 0.424, 95% CI = 0.210–0.854, P = 0.010) patients.

**Table 3:** Multivariable analysis for osteoporosis at the end of follow-up by using Cox regression.

Variables^a^	Crude HR	95% CI	P-value	Adjusted HR	95% CI	P-value
PBC^a^	3.225	2.677–3.886	<0.001*	3.333	2.712–4.098	<0.001*
**Gender**						
Male^b^	0.494	0.418–0.585	<0.001	0.567	0.419–0.678	<0.001*
**Age groups**						
30–39^c^	0.488	0.181–1.321	0.158	0.524	0.182–1.389	0.192
40–49^c^	0.982	0.423–2.281	0.966	1.111	0.460–2.510	0.787
50–59^c^	0.735	0.323–1.673	0.463	0.916	0.382–2.142	0.811
≧60^c^	0.963	0.430–2.155	0.927	1.270	0.595–2.694	0.542
LC^a^	1.179	0.890–1.562	0.252	0.757	0.527–1.013	0.064
HTN^a^	0.608	0.502–0.736	<0.001*	0.611	0.436–0.786	<0.001*
DM^a^	0.704	0.580–0.855	<0.001*	0.827	0.644–1.035	0.097
CHF^a^	0.416	0.264–0.656	<0.001*	0.499	0.312–0.795	0.001*
Stroke^a^	0.733	0.549–0.981	0.037*	0.811	0.601–1.133	0.382
CAD^a^	0.636	0.474–0.853	0.002*	0.795	0.534–1.075	0.127
Hyperlipidemia^a^	0.751	0.476–1.185	0.218	1.030	0.612–1.608	0.845
Dementia^a^	1.015	0.598–1.723	0.956	0.812	0.425–1.332	0.411
CKD^a^	0.475	0.372–0.688	<0.001*	0.459	0.317–0.754	<0.001*
RA^a^	3.280	1.998–5.385	<0.001*	2.111	1.301–3.989	<0.001*
Postmenopausal^a^	4.304	1.074–17.246	0.039*	3.012	0.746–12.001	0.198
COPD^a^	0.958	0.735–1.249	0.752	0.960	0.712–1.144	0.565
Sjögren’s syndrome^a^	4.624	2.395–8.926	<0.001*	1.945	0.897–3.542	0.188
RTA^a^	0.481	0.240–0.966	0.040*	0.424	0.210–0.854	0.010*
Steroid use^a^	0.769	0.344–1.717	0.521	0.738	0.304–1.608	0.457
UDCA use^a^	0.867	0.452–1.226	0.721	0.851	0.446–1.211	0.633
Denosumab use ^a^	1.125	0.164–1.756	0.288	1.045	0.198–1.562	0.385
SERMs ^a^	0.976	0.477–1.579	0.714	1.000	0.420–1.411	0.785
Bisphosphonate use ^a^	1.045	0.386–1.970	0.597	1.052	0.355–2.043	0.583
Hip fracture^a^	3.154	2.161–4.604	<0.001*	2.267	1.542–3.796	<0.001*
Wrist fracture^a^	1.416	0.780–2.570	0.253	0.912	0.487–1.702	0.733
Vertebral fracture^a^	9.425	7.272–12.217	<0.001	6.904	5.101–8.999	<0.001*
Rib fracture^a^	0.723	0.232–2.246	0.575	0.735	0.235–2.340	0.598
CCI_R	0.927	0.888–0.969	0.001*	0.922	0.866–0.989	0.001*

HR = hazard ratio, CI = confidence interval, Adjusted HR: Adjusted for all the variables listed in the table.

*P-value <0.05.

^a^.Without the disease or medication as reference.

^b^.Female as reference.

^c^.Age 18–29 year old as reference.

CCI_R denotes Charlson comorbidity index removed DM, CHF, stroke, COPD, and liver diseases.

**Fig 2:**
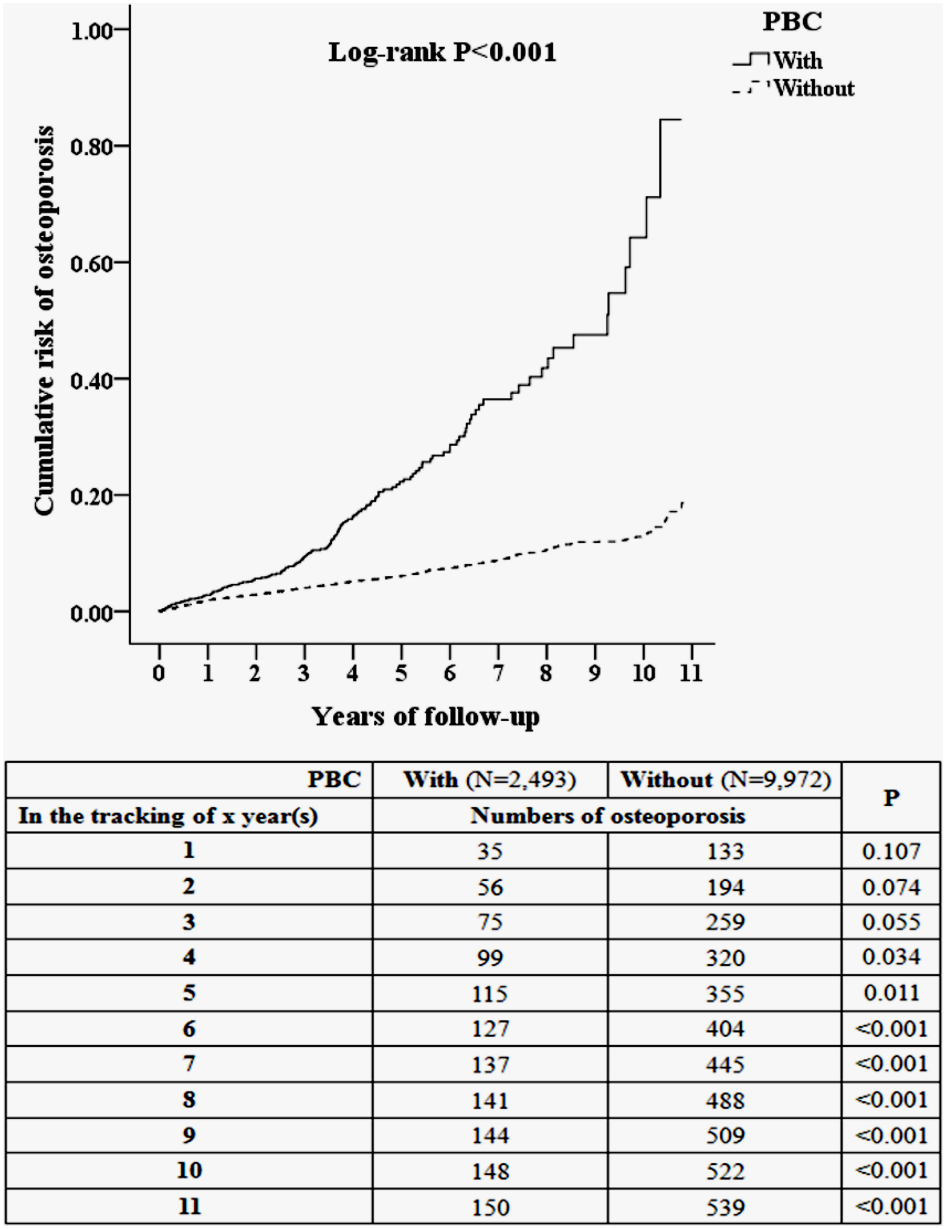
Kaplan-Meier for cumulative risk of osteoporosis among aged 18 and over stratified by PBC with log-rank test.

Fig 2 compares the Kaplan–Meier curves for the cumulative incidence of osteoporosis between the PBC and non-PBC cohorts after 11 years of follow-up. The 1-, 5-, and 11-year actuarial rates of osteoporosis were 1.40%, 4.61%, and 6.01% in the PBC cohorts and 1.36%, 3.55%, and 5.40% in the non-PBC cohorts, respectively.

We found that that 150 (6.02%) PBC cohort members progressed to osteoporosis with 10,570 person-years of follow-up over 11 years, for an incidence rate of 1,418 per 100,000 person-years. Conversely, only 539 (5.41%) of the non-PBC cohort members progressed to osteoporosis over the 49,998 person-years of follow-up for 11 years, for an incidence rate of 1,078 per 100,000 person-years. Therefore, the incidence rate of osteoporosis was 1.31-fold higher in PBC cohort than in the non-PBC cohort.

After stratification, the risk of osteoporosis notably increased independent of status regarding liver cirrhosis, hypertension, CHF, stroke, CAD, hyperlipidemia, DM, or CKD. Additionally, older age was associated with an increasing risk of osteoporosis in the PBC cohort. Remarkably, UDCA use and bone anti-resorptive agents (denosumab, SERMs, bisphosphonate) had no significance in decreasing the risk of osteoporosis in the PBC cohort (Table 4).

**Table 4:** Factors of osteoporosis at the end of the follow-up period stratified by Cox regression.

Variables	PBC			Non PBC			Ratio	Adjusted HR (95%CI)	P-value
Osteoporosis	Event	PYs	Rate	Event	PYs	Rate			
	150	10,570	1,418.99	539	49,998.72	1,078.03	1.316	3.333 (2.712–4.098)	<0.001*
Gender									
**Male**	**42**	**4,090.61**	**1,026.74**	**145**	**21,655.97**	**699,56**	**1.533**	**3.342** **(2.831–4.764)**	<0.001*
**Female**	**108**	**6,480.30**	**1,666.59**	**394**	**28,342.75**	**1,390.13**	**1.199**	**3.155** **(2.224–4.017)**	<0.001*
Age, year									
18–29	2	127.04	1,574.31	4	374.21	1,068.92	1.473	3.485 (0.424–26.121)	0.238
**30–39**	**5**	**406.77**	**1,229.20**	**6**	**1,457.81**	**411.58**	**2.987**	**21.395** **(3.627–135.841)**	<0.001*
**40–49**	**18**	**1,316.49**	**1,367.27**	**37**	**3,436.19**	**1,076.77**	**1.270**	**3.751** **(1.984–6.912)**	<0.001*
**50–59**	**34**	**2,572.05**	**1,321.90**	**74**	**9.456.33**	**782,54**	**1.689**	**3.201** **(2.010–5.124)**	<0.001*
**≧60**	**91**	**6,148.56**	**1,480.02**	**418**	**35,274.18**	**1,185.00**	**1.249**	**2.998** **(2.341–3.812)**	<0.001*
Comorbidity									
LC	37	3,208	1,153.16	16	2,186.22	731.86	13576	3.782 (1.844–7.628)	<0.001*
HTN	29	1,593.93	1,89.40	101	14,325.91	705.02	2.581	8.712 (5.412–13.875)	<0.001*
DM	27	1,911.66	1,412.39	98	12,164.48	805.62	1.753	4.951 (2.875–7.897)	<0.001*
CHF	3	296.32	1,012.42	16	3,333.08	480.04	2.109	12.012 (2.512–54.124)	<0.001*
Stroke	13	568.73	2,285.79	36	4,907.65	733.55	3.116	11.101 (5.044–23.012)	<0.001*
CAD	8	588.18	1,360.13	40	5,518.99	724.77	1.877	5.956 (2.344–14.845)	<0.001*
Hyperlipidiemia	6	179.59	3,340.94	13	1,900.49	684.03	4.884	19.0245 (4.524–98.724)	<0.001*
CKD	6	891.28	673.19	23	4,225.80	544.28	1.237	3.452 (1.211–10.682)	<0.001*

PYs = Person-years; Rate: per 10^5^ PYs; Ratio = rate in cases÷ Rate in controls; Adjusted HR: Adjusted for all the variables listed in Table 3. CI = confidence interval.

*P-value <0.05.

Because females predominate in PBC cases, we further isolated the female patients with PBC using stratification and found the same trend as with increasing age (Table 5).

**Table 5:** Factors of osteoporosis among female patients stratifies by age groups by using Cox regression.

	PBC			Non PBC					
	Event	PYs	Rate (per 10^5^ PYs)	Event	PYs	Rate (Per 10^5^ PYs)	Ratio	Adjusted HR 95% CI	P
Female	108	6,480.30	1,666.59	394	28,342.75	1,390.13	1.199	3.155 (2.224–4.017)	<0.001*
Age, years									
**18–29**	**1**	**73.56**	**1,359.43**	**1**	**192.18**	**520.35**	**2.613**	**8.159** **(0.897–24.512)**	**0.887**
**30–39**	**1**	**187.73**	**532.68**	**0**	**758.73**	**0.00**	-	-	-
**40–49**	**8**	**641.09**	**1,247.87**	**10**	**1,351.86**	**739.72**	**1.687**	**6.424** **(1.988–19.542)**	0.001*
**50–59**	**29**	**1,710.67**	**1,695.24**	**39**	**4,532.53**	**860.45**	**1.970**	**5.014** **(2.912–8.842)**	<0.001*
**≧60**	**69**	**3,867.25**	**1,784.21**	**344**	**21,507.45**	**1,599.45**	**1.116**	**2.975** **(2.041–4.182)**	<0.001*

PYs = Person-years; Rate: per 10^5^ PYs; Ratio = rate in cases÷ Rate in controls; Adjusted HR: Adjusted for all the variables listed in Table 3. CI = confidence interval.

*P-value <0.05.

**Fig 3:**
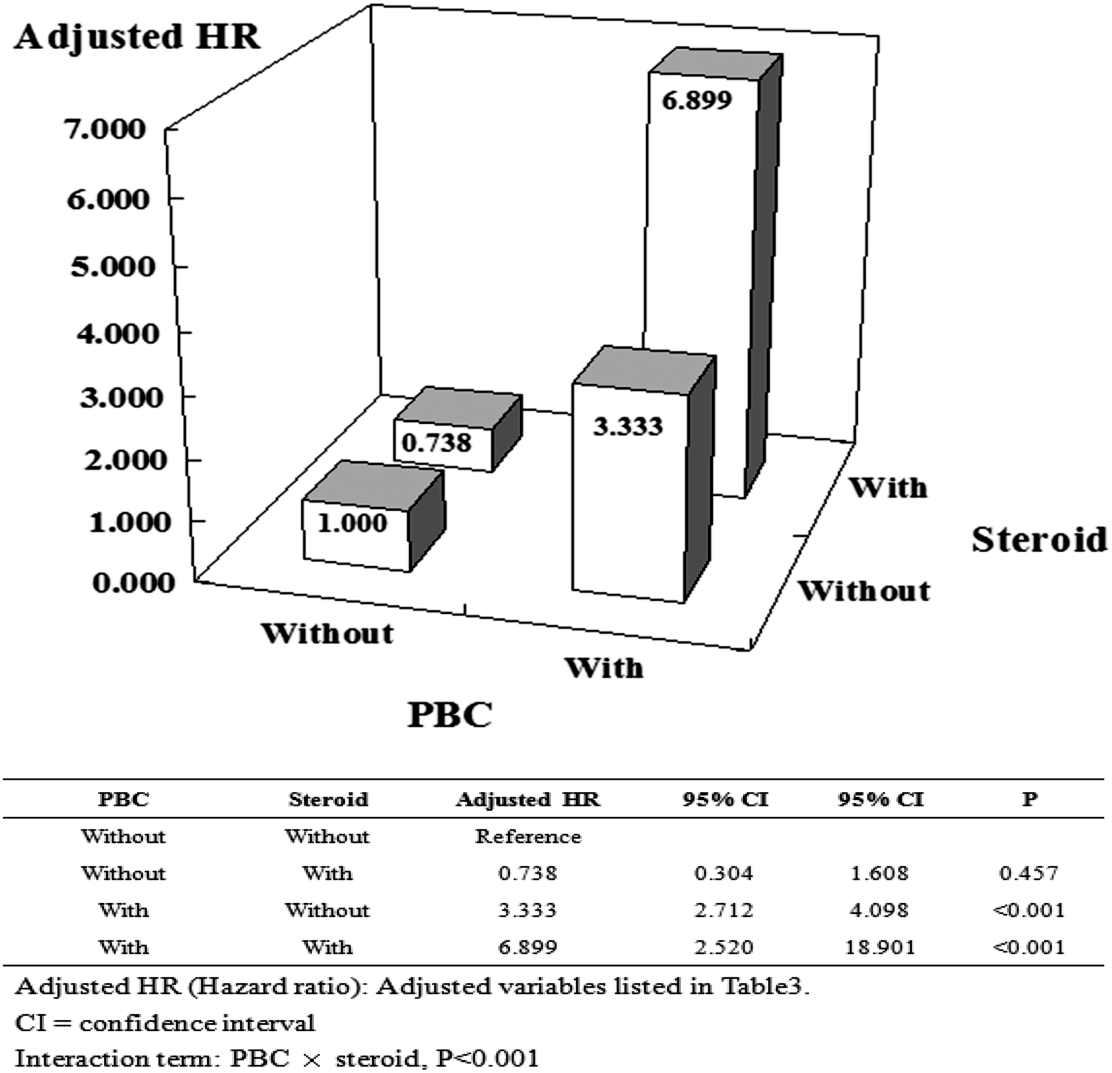
Factors of osteoporosis stratified by PBC and steroid.

To further explore the risk of osteoporosis with the use of steroids in the treatment of PBC, we limited the factors influencing osteoporosis and stratified only by PBC and steroid use (Fig 3). The results indicated that steroid use will aggravate the risk of osteoporosis when comparing PBC to non-PBC cohorts (aHR: 6.899 vs 3.333).

Additionally, to elucidate the influence of liver cirrhosis on PBC and the risk of osteoporosis, we further limited the factors influencing osteoporosis and stratified by cirrhotic PBC versus non-cirrhotic PBC and found that the non-cirrhotic patients are prone to osteoporosis at a younger age (Tables 6 and 7).

**Table 6:** Factors of osteoporosis among liver cirrhosis patients by variable listed in the table by Cox regression.

Variables	PBC			Non PBC			Ratio	Adjusted HR (95%CI)	P-value
Osteoporosis	Event	PYs	Rate	Event	PYs	Rate			
	37	3,208.58	1,153.16	16	2,186.22	731.86	1.576	3.782 (1.844–7.628)	<0.001*
Gender									
**Male**	**14**	**1,461.70**	**957.79**	**5**	**1,188.40**	**420,73**	**2.276**	**4.411** **(1.254–15.642)**	0.010*
**Female**	**23**	**1,746.88**	**1,316.63**	**11**	**997.82**	**1,102.40**	**1.194**	**2.987** **(1.165–7.424)**	0.008*
Age, year									
18–29	0	65.77	0,00	0	2.39	0,00	-	- -	-
**30–39**	**2**	**207.19**	**956.30**	**0**	**2.39**	**0.00**	-	- -	-
**40–49**	**7**	**474.91**	**1,473.96**	**2**	**183.99**	**1,087.02**	**1.356**	**3.945** **(0.322–39.842)**	**0.265**
**50–59**	**7**	**885.52**	**790.50**	**2**	**485.50**	**411,95**	**1.919**	**5.598** **(0.424–70.943)**	**0.199**
**≧60**	**21**	**1,575.19**	**1,333.17**	**12**	**1,502.70**	**798.56**	**1.669**	**3.685** **(1.511–8.572)**	<0.001*

PYs = Person-years; Rate: per 10^5^ PYs; Ratio = rate in cases÷ Rate in controls; Adjusted HR: Adjusted for all the variables listed in Table 3. CI = confidence interval.

*P-value <0.05

**Table 7:** Factors of osteoporosis among without liver cirrhosis patients by variable listed in the table by Cox regression.

Variables	PBC			Non PBC			Ratio	Adjusted HR (95%CI)	P-value
Osteoporosis	Event	PYs	Rate	Event	PYs	Rate			
	113	4,362.33	1,534.84	523	247,812.50	1,093.86	1.403	3.249 (2.652–4.123)	<0.001*
Gender									
**Male**	**28**	**2,628.90**	**1,065.08**	**140**	**20,467.57**	**684,01**	**1.557**	**3.156** **(2.239–4.011)**	<0.001*
**Female**	**85**	**4,733.43**	**1,795.74**	**383**	**27,344.93**	**1,400.63**	**1.282**	**3.421** **(2.705–5.978)**	<0.001*
Age, year									
18–29	2	61.27	3,264.24	4	371.83	1,075.76	3.034	11.121 (0.944–98.240)	0.068
30–39	3	199.58	1,503.16	6	1.446.17	414.89.	3.623	25.345 (0.702–645.644) -	0.073
**40–49**	**11**	**841.58**	**1,307.07**	**35**	**3,252.20**	**1,076.19**	**1.215**	**4.088** **(1.901–8.512)**	<0.001*
**50–59**	**27**	**1,686.54**	**1,600.91**	**72**	**8,970.83**	**802,60**	**1.995**	**3.877** **(2.251–6.972)**	<0.001*
**≧60**	**70**	**4,573.36**	**1,530.60**	**406**	**33,771.47**	**1,202.20**	**1.273**	**3.171** **(2.401–4.599)**	<0.001*

PYs = Person-years; Rate: per 10^5^ PYs; Ratio = rate in cases÷ Rate in controls; Adjusted HR: Adjusted for all the variables listed in Table 3. CI = confidence interval.

*P-value <0.05

**Fig 4:**
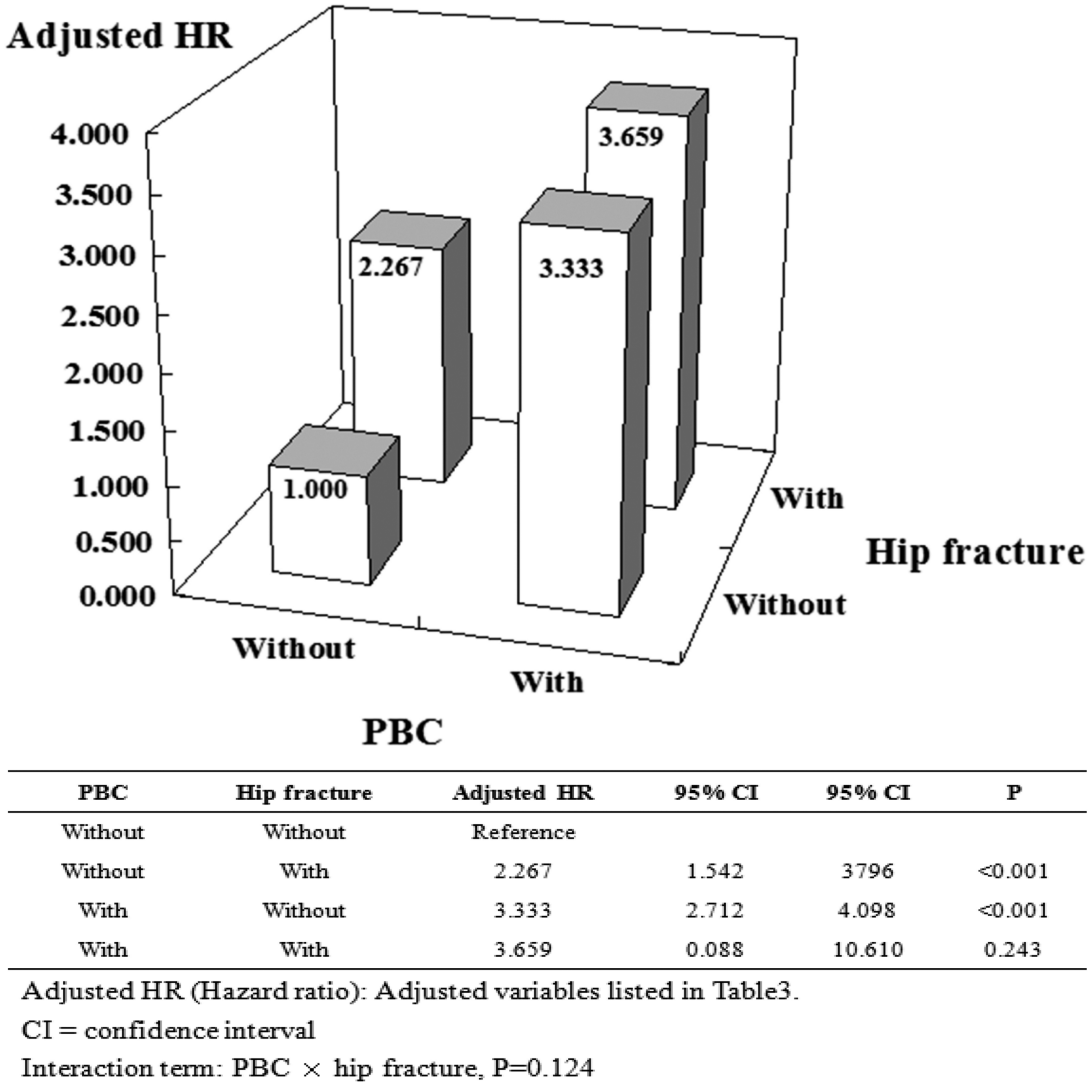
Factors of osteoporosis stratified by PBC and hip fracture.

**Fig 5:**
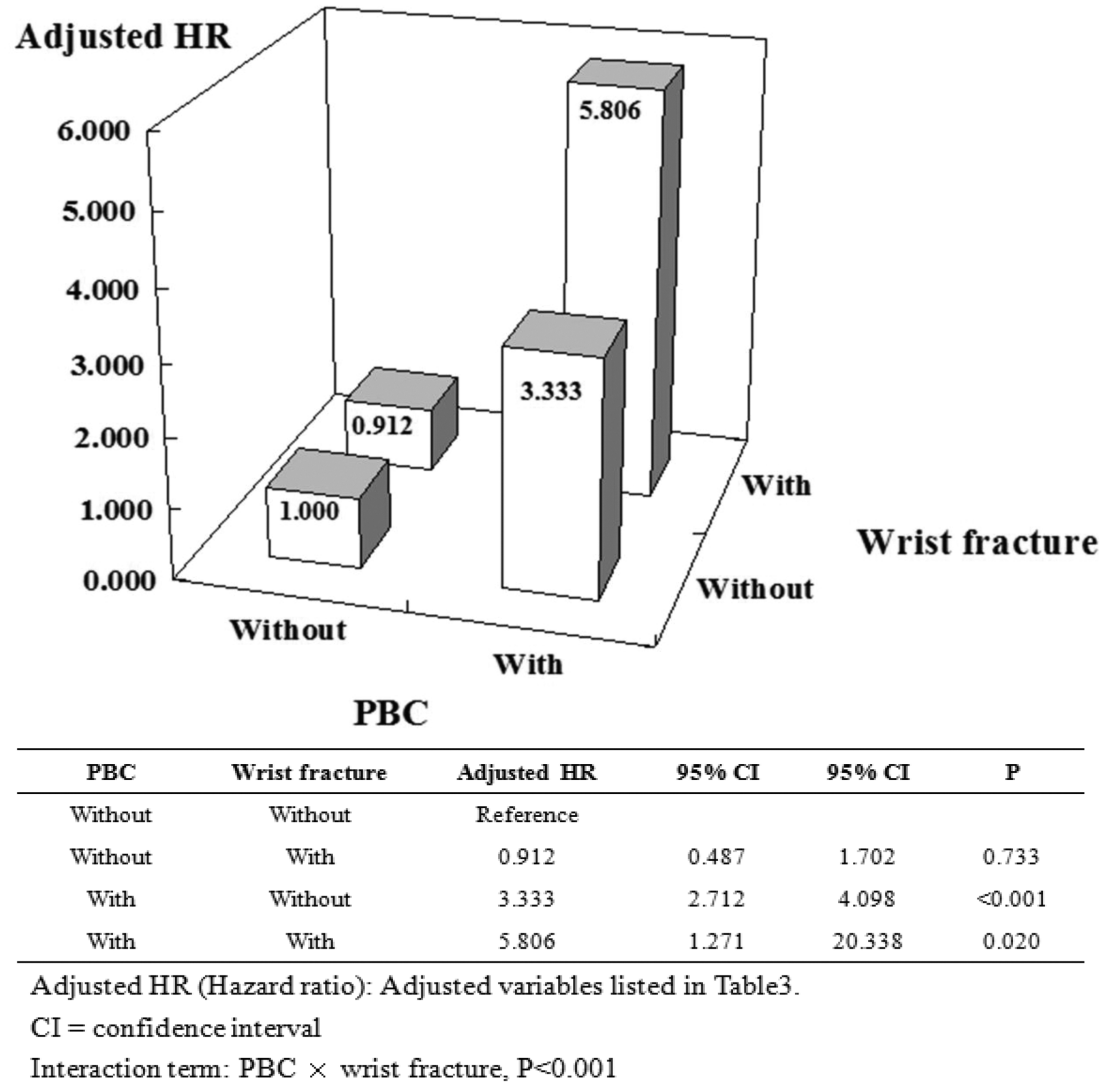
Factors of osteoporosis stratified by PBC and wrist fracture.

**Fig 6:**
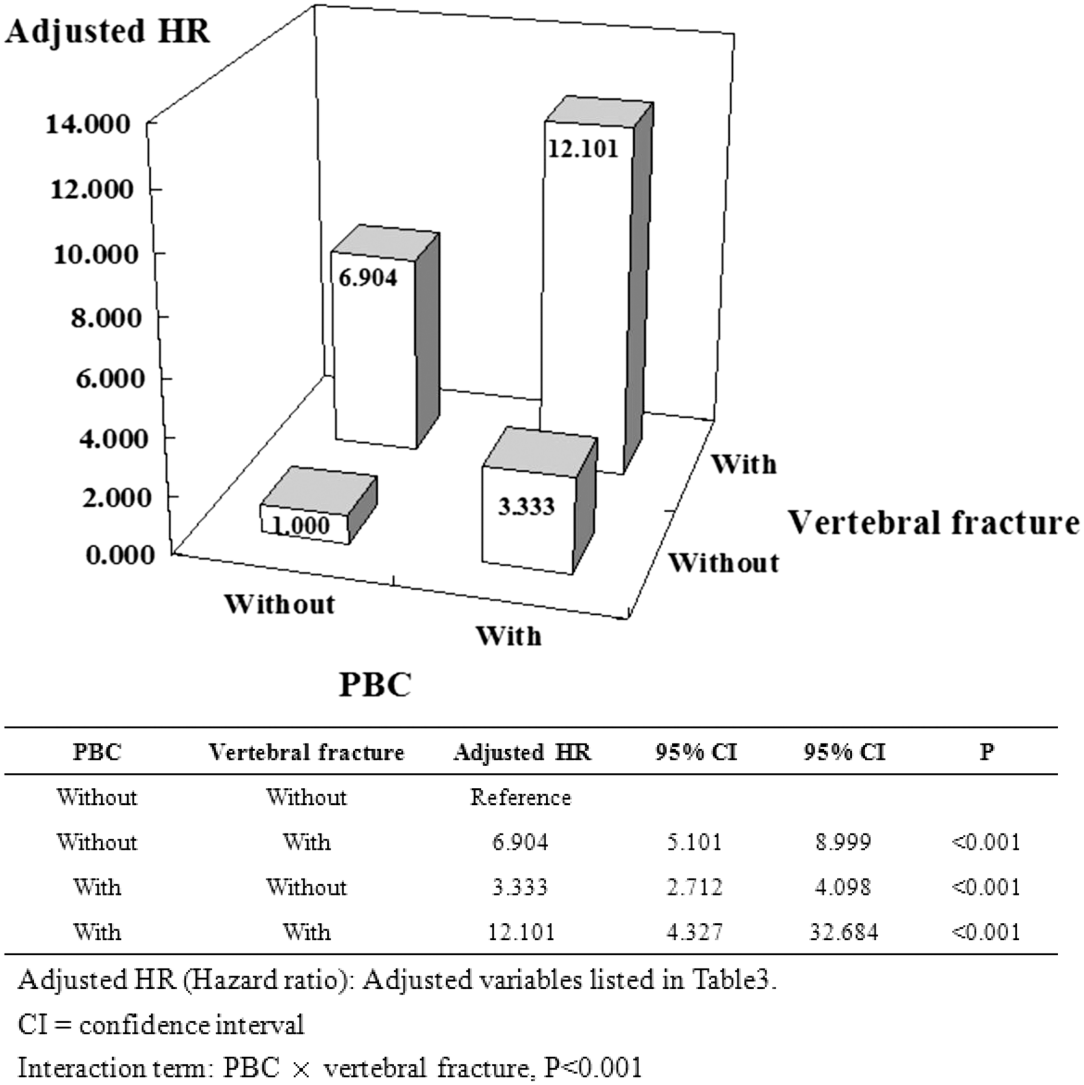
Factors of osteoporosis stratified by PBC and vertebral fracture.

To clarify the risk of various fractures between PBC and non-PBC cohorts we held constant osteoporosis, together with its explanatory variables, and stratified by PBC status as well as several prominent types of fractures. Using non-PBC without hip fracture as a reference, the association was increased in solely PBC cohorts and solely hip fracture cohorts (aHR: 3.333 vs 2.267); however, there was remarkably no associated increase in PBC with hip fractures. Likewise, using non-PBC without wrist fracture as a reference, the association was increased in solely PBC cohorts and PBC with wrist fracture (aHR: 3.333 vs 5.806); however, we found no associated increase with solely wrist fracture (Figs 4 and 5). Finally, using non-PBC without vertebral fracture as a reference, the association was increased in solely PBC cohorts and in the solely vertebral fracture cohorts, with a greater increased PBC in the vertebral fracture cohorts (aHR: 3.333 vs 6.904 vs 12.101) (Fig 6).

## Discussion

In this study, PBC was associated with a 3.33-fold increase in the risk of osteoporosis compared with non-PBC cohorts after adjusting for numerous potential confounders. The results are compatible with previous reports regarding female predominance (56.96%) and a relative low prevalence (6.02%) of the PBC cohorts progressing to osteoporosis over the 11 years of follow-up. In the baseline demography, we found that liver cirrhosis, hyperthyroidism, Sjogren’s syndrome, and renal tubular acidosis are common comorbidities associated with PBC.

Although the underlying mechanism of osteoporosis remains unclear; metabolic imbalances caused by osteoprotegerin (OPG)-receptor activator of nuclear factor-κB ligand (RANKL) was one of the leading mechanisms proposed for bone remodeling. [12] The process of liver cirrhosis, including PBC may disrupt the osteoblasts functing by decreasing the production of growth factors, such as insulin-like growth factor-1, increasing the synthesis of oncofetal fibronectin, and decreasing blood levels of osteocalcin. Some in vitro studies have demonstrated that unconjugated bilirubin and lithocholic acid contribute to the toxic effect on bone precursors and osteoblasts during the progress of cirrhosis.[13] In addition, vitamin-K deficiency, frequently observed in cholestasis, known to impair the osteoclast maturation and function, whereas vitamin-D deficiency might cause secondary hyperparathyroidism, which increases the bone resorption and extends the deficit of calcium ions. [14,15] After analyzing osteoporosis stratified by various comorbidities using Cox regression models, we found that in comparing PBC to non-PBC cohorts, older age is associated with increasing risk of osteoporosis in PBC. However, UDCA use remarkably had no significance after stratification in decreasing the risk of osteoporosis in PBC cohorts. Agents used to treat liver disease may also affect bone mass. Our study showed that the use of steroids and UDCA have no beneficial effect in decelerating the process of osteoporosis, which is consistent with a previous study. [16,17,18]In our further cross section analysis, steroids increased the risk of osteoporosis in PBC cohorts by approximately 2-fold compared with no steroid use (Fig 3). Steroid use is not a common regime in PBC therapy despite its productivity in autoimmune hepatitis and with other immunosuppressive therapies after liver transplantation.[19]

Trabecular bone loss reportedly accelerated after 12 months use of more than 7.5 mg/day of prednisone. Corticosteroids increase osteoclast differentiation and activity by the production of interleukins, specifically IL-1 and IL-6, and decrease osteoblast differentiation by suppressing differentiation, recruitment, and indirectly reducing collagen synthesis.

Moreover, increased bone resorption is more evident in cholestatic women.[20]The estrogen deficiency state in postmenopausal females has been proposed as a possible mechanism of osteoporosis in PBC women.[6]However, our study notably showed no close relationship between postmenopausal state and osteoporosis in PBC patients, in contrast to results obtained in previous studies of this topic. This may be because we are unable to fully identify the early postmenopausal or late postmenopausal states. Benetti et al reported that early postmenopausal (less than 5 years) PBC patients had a 6.5 times greater risk of having osteoporosis than those with premenopausal PBC, whereas the late postmenopausal (more than 5 years) patients had a remarkable 9.6 times greater risk.[21]Notably, other retrospective studies have obtained results consistent with our findings, showing that menopausal status is not a significant independent factor for the development of osteoporosis in PBC patients and that it appears to have an effect far less strong than the severity of liver damage.[22]

In our study liver cirrhosis is also correlated with osteoporosis in PBC. Cirrhosis has been linked to increased risk of fracture by approximately 2-fold, compared to non-cirrhotic liver diseases, including PBC.[23]Bone density in patients diagnosed with PBC before developing a cirrhotic state is like that of healthy controls. Among patients with cirrhosis, other variables, such as a severely clinical Child-Pugh or Mayo Risk Score, histological stage (Ludwig, Sheuer), and lower BMI, showed progressive correlation with low BMD.[24]We compared the liver cirrhosis and non-cirrhosis cohorts with PBC and found that the non-cirrhosis PBC cohorts are prone to osteoporosis at a younger age than the cirrhosis PBC cohorts (Tables 6 and 7). According to a large retrospective study by Seki et al with 128 PBC postmenopausal women, even non-cirrhosis PBC patients were found to have a higher risk of osteoporosis. The study concluded that the mechanism is predisposed by low bone turnover manifested via lobular cholestasis in non-cirrhotic PBC stages observed via a histological examination.[25]In both the non-cirrhotic and cirrhotic PBC cohorts, we found female predominance and speculated that the reason non-cirrhotic PBC patients are prone to osteoporosis at a younger age compared to the cirrhotic PBC cohort may be related to different hormone conditions.

Finally, we also analyzed the association of fracture potential between the PBC and non-PBC cohorts and found a strong osteoporosis risk for PBC patients in the vertebral fracture cohort. However, this result is inconsistent with a previous study that showed a 2-fold increase in the risk of any fracture for the PBC cohort compared with the general population.[26]The fracture risk with PBC remains widely debated.

There are several limitations to our study. First, the health insurance data we utilized did not include laboratory results such as bilirubin level, alkaline phosphatase level, lifestyle data, exercise capacity, body weight, body mass index, nutrition supplements, and family history of systemic disease. It also excluded disease details such as the histological stage of liver biopsy; severity of the PBC; intake of calcium and vitamin D; or the use of bisphosphonate, hormone therapy, and calcitonin.

Second, the inclusion of disease attributed to alcohol and tobacco in our analysis could underestimate the results for these individuals.

Third, the background of the patients in this study was predominantly Asian, limiting the generalizability of these results.

## Conclusions

In conclusion, PBC patients are at high risk of osteoporosis, and medications involving glucocorticoids and the status of post liver cirrhosis involvement may play a crucial role in the occurrence of osteoporosis in PBC patients. Nonetheless, further study is required to fully elucidate the pathophysiology, treatment, and prevention of osteoporosis in PBC patients.

## Abbreviations

BMD: bone mineral density
CAD: coronary artery disease
CCI: charlson comorbidity index
CHF: congestive heart failure
CHF: congestive heart failure
CI: confidence interval
CKD: chronic kidney disease
COPD: chronic obstructive pulmonary disease
DM: diabetes mellitus
HR: hazard ratio
IBD: inflammatory disease
IRR: incidence rate ratio
LC: liver cirrhosis
NHI: The National Health Insurance
NHIRD: The National Health Insurance Research Database
PBC: primary biliary cirrhosis
RA: rheumatoid arthritis
RTA: renal tubular acidosis
UDCA: ursodeoxycholic acid
